# The Sensory Quality and the Textural Properties of Functional Oolong Tea-Infused Set Type Yoghurt with Inulin

**DOI:** 10.3390/foods10061242

**Published:** 2021-05-29

**Authors:** Katarzyna Świąder, Anna Florowska, Zuzanna Konisiewicz

**Affiliations:** 1 Department of Functional and Organic Food, Institute of Human Nutrition Sciences, Warsaw University of Life Sciences (SGGW–WULS), 159C Nowoursynowska Street, 02-787 Warsaw, Poland; zuzanna.konisiewicz@gmail.com; 2 Department of Food Technology and Assessment, Institute of Food Science, Warsaw University of Life Sciences (SGGW–WULS), 159C Nowoursynowska Street, 02-787 Warsaw, Poland; anna_florowska@sggw.edu.pl

**Keywords:** functional food, yoghurt, tea, oolong tea, inulin, sensory quality, consumer test, texture properties, food design

## Abstract

Set type yoghurts are characterised by a semi-solid texture, which is created during the fermentation process. The tea infusion in this type of yoghurt production can influence the quality of the final product. Therefore, the aim of the experiment was to evaluate the influence of the addition of 3, 6 and 9% inulin to oolong tea-infused yoghurts on the sensory quality. It has been evaluated by trained experts using a Quantitative Descriptive Profile analysis and by consumers using hedonic scaling, as well as on instrumentally evaluated features such as texture, stability and visual parameters. The addition of oolong tea to yoghurt resulted in positive changes in the perception of sweet, peach and nectar odours and flavours, and also creaminess, as well as negative changes in the presence of a bitter taste, the whey presence and a colour intensification towards dark cream (*p* ≤ 0.05). The addition of inulin to the tested oolong tea yogurts caused a decrease in the whey presence and brightened the yoghurt’s colour (6% and 9%, *p* ≤ 0.05, respectively), as well as an improved creaminess and an increase in the sweet taste of the yoghurt. It was also observed that the addition of oolong tea deteriorated the instrumentally evaluated texture of the set yoghurts, while inulin at a higher concentration (9%, *p* ≤ 0.05) increased the firmness and adhesiveness. Moreover, the addition of inulin also had a positive effect on the yoghurt’s stability. The addition of inulin to oolong tea-infused set yoghurts may be valuable both as a source of prebiotic fibre in functional products and as a factor improving the quality of these products.

## 1. Introduction

Due to growing interest of consumers in high quality functional food products, it is very important to design new food products with health and nutritional claims and a clean label, which determines the use of natural food ingredients, but also products that, at the same time, have a high sensory quality and are acceptable to consumers.

An important challenge in food design is the development of a product that meets the expectations of the consumers to whom it is addressed and thus becomes commercially viable [1,2]. Various methods of sensory analysis are used to investigate consumers’ expectations of a designed product, their reaction to it and to determine its quality [2], and these are applied at different stages of food product development (NPD) [3,4,5,6,7]. However, in this NPD process, it is important to remember that the value of the product to the consumer must be clearly explained [8]. This is extremely important in the design of functional foods.

However, which products can be called functional foods? Based on the definition provided by Diplock et al. [9], “A food can be regarded as functional if it is satisfactorily demonstrated to affect beneficially one or more target functions in the body, beyond adequate nutritional effects, in a way that is relevant to either improved stage of health and well-being and/or reduction of risk of disease. A functional food must remain food and it must demonstrate its effects in amounts that can normally be expected to be consumed in the diet: it is not a pill or a capsule, but part of the normal food pattern”.

According to Roberfroid [10], functional food should present the following characteristics: “1. a conventional or everyday food, 2. consumed as part of the normal/usual diet, 3. composed of naturally occurring (as opposed to synthetic) components, perhaps in unnatural concentrations or present in foods that would not normally supply them, 4. having a positive effect on target function(s) beyond nutritive value/basic nutrition, 5. that may enhance well-being and health and/or reduce the risk of disease or provide health benefit so as to improve the quality of life including physical, psychological and behavioural performances, and 6. have authorized and scientifically based claims”.

In our research, in order to develop a functional product, we focused on the following products, i.e., natural yoghurt, oolong tea and inulin, which meet the above-mentioned criteria.

Yoghurt is a product rich in nutrients, in addition to being a low energy density food [11], which owes its properties to the presence of live bacteria and proteins, as well as lipids, vitamins and minerals [12,13]. For yoghurts containing live yoghurt cultures of *Lactobacillus delbruecki* subsp. *bulgaricus* and *Streptococcus thermophilus*, the European Food Safety Authority (EFSA) has endorsed a health claim that yoghurt cultures help to improve lactose digestion, which has a beneficial physiological effect on people with lactose maldigestion syndrome [14].

We chose yoghurt as one of the most frequently consumed functional products in the world [15,16]. It is often consumed as part of an everyday diet especially in France and Germany (the countries with the highest overall consumption volumes of yoghurt), but the highest per capita yoghurt consumption in 2015–2017 was reported in Sweden (33.4 kg) and the Netherlands (30.5 kg) [17]. Outside Europe, yoghurt consumption is also growing in all Asian countries. The highest per capita yoghurt consumption between 2015 and 2017 was recorded in Japan (6.2 kg), South Korea (3.2 kg) and Thailand (1.6 kg), while consumption of this nutritionally valuable product is almost non-existent in other Asian countries [17]. According to forecasts [18], the average consumption of yoghurt in the world will reach 7.3 kg in 2021 and the yoghurt segment of the dairy market is expected to increase by 2.3% in 2022. The highest revenue in the yoghurt segment, considering the global market, is generated in China [18]. China is also the world leader in tea production, with tea consumption reaching 0.6 kg per person per year. However, this is not the highest value in the world. The leader in tea consumption is Turkey, where tea consumption is around 3.2 kg per capita per year, according to 2016 data [19]. Tea, after water, is the most consumed beverage in the world [19,20,21,22]. Its consumption worldwide in 2020 was 6.3 billion kg, and by 2025 it is forecasted to reach 7.4 billion kg [19].

Tea contains over 4000 chemical compounds, some of which have health-promoting properties [23,24]. Due to the fermentation process that *Camellia sinensis* leaves undergo, tea can be divided into fermented (black tea), semi-fermented (oolong tea) and unfermented (green) [20,25,26], best known for its health-promoting properties [27]. In 2018, the EFSA [27] assessed the safety of green tea catechins from dietary sources (infusions and food supplements) following concerns about their potential harmful effects on the liver. Based on the EFSA opinion [27], it can be concluded that catechins from green tea infusions and similar beverages are safe, while in the case of dietary supplements, the daily intake of catechins must not exceed 800 mg/day because, at this level, they may pose a health risk. Black tea (fermented) and oolong tea (semi-fermented) contains a mixture of catechins and their oxidised substances, (i.e., theaflavins and thearubigins) unlike green tea, which contains monomeric catechins (i.e., EC, ECG, EGC and EGCG) [24,28]. Oolong, in addition to its health promoting properties resulting from the presence of the above-mentioned substances, is characterized by an interesting peach–nectarine flavour and aroma and lacks the intense bitterness that is characteristic of green tea [20].

The addition of tea to yoghurt changes the sensory quality of the yoghurt, both in taste and texture [20], and the use of inulin, known for its texture-enhancing properties in food products [29,30,31], could help in this process.

Inulin belongs to the fructan-type polysaccharide [32] and occurs in high concentrations in chicory root (*Chicorium intybus*) [33]. The addition of “native chicory inulin” into a food product allows for the use of the following health claim: “Chicory inulin contributes to normal bowel function by increasing stool frequency”. In fact, 12 g of “native chicory inulin” should be consumed daily to achieve the desired health effect [33]. Additionally, for products that contain at least 6 g of fibre per 100 g, the nutrition claim may be made that the product is high in fibre [34]. Due to its functional and health-promoting properties, i.e., its function as a dietary fibre, its prebiotic effect and its reduced calorific value, inulin is increasingly used in the food [29] and pharmaceutical industries [32]. Inulin is also used as a protein stabiliser and as a diagnostic tool for kidney function [32].

Various studies have assessed the effect of inulin on yoghurt’s sensory qualities [35,36,37,38,39,40], and there are also studies describing the properties of tea-infused yoghurt as evaluated by consumers [41,42,43,44]. In our recent study, the effect of different kinds of tea (green tea, black tea, oolong tea and lemon balm) on yoghurt quality were analysed [20] and, to the best of our knowledge, there are no studies assessing the effect of inulin on the textural and sensory properties of oolong tea-infused yoghurt. Therefore, the main objective of this study was to investigate the effect of inulin addition on the sensory quality and textural properties of oolong tea-infused yoghurt, that would allow for the next steps in the process of designing functional products to take place.

## 2. Materials and Methods

### 2.1. Materials

The research material consisted of six types of yoghurt. The yoghurts were prepared using a thermostatic method from pasteurised and microfiltered cow’s milk with a fat content of 3.2% (Piatnica, Poland). Freeze-dried starter cultures YO-122 (Serowar, Szczecin, Poland) were used to inoculate the milk. The starter cultures contained *Streptococcus salivarius* subsp. *thermophilus* and *Lactobacillus delbrueckii* subsp. *bulgaricus*. For the Oolong tea (*Camellia sinensis*), a type of Oolong milky leaf tea (Herbaty Szlachetne Sp. Z o. o., Szczecin, Poland) was used to produce a tea-infused yoghurt. Frutafit^®^ CLR inulin (inulin ≥85% dm, sweetness 30%, chicory root, DP 2–10) (Sensus, Roosendaal, The Netherlands) was used to enrich the yoghurt.

#### Yoghurt Processing

A yoghurt was developed according to the methodology described by Świąder et al. [20]. The raw ingredients for yoghurt production were weighed using an analytical balance (PS 1000/C/2, Radwag, Radom, Poland). To prepare the tea-infused yoghurt, the milk was first heated for 30 min to 85 °C, infused with oolong tea leaves (2 g tea/100 mL milk) and steeped for 10 min under a cover. The infusion, manually filtered through gauze strainers, was cooled. Then, starter cultures (0.1%) were added. The sample was poured in the amount of 100 mL into transparent, plastic and sterile containers with lids. The samples were then thermostated at 43 °C for about 4.5 h in an incubator (INE 500, Memmert, Schwabach, Germany) until they reached a pH value of 4.5–4.6 (Voltcraft PH-100ATC, Conrad Electronic Sp. z.o.o., Wrocław, Poland). Afterwards, the samples were cooled and then stored at 4 °C until the structure was built. In order to obtain yoghurts with inulin, before the heating process, inulin, in the amounts of 3, 6 and 9%, was additionally added to three milk samples, respectively, and no tea was added to another to obtain natural yoghurt as a control sample.

### 2.2. Methods

#### 2.2.1. Sensory Analysis

*The Expert Test Method:* In order to obtain an objective assessment of the quality of the produced yoghurts, the Quantitative Descriptive Profile (QDP) method was used. According to the procedure described in ISO 13299:2016 [45], 33 sensory descriptors were selected and defined (Supplementary Material-Table S1) [20]. Among them, eight described the odour of the yoghurts evaluated (sweet, sour, yoghurt, milky, fat, peach, nectar and citrus), seven described the external appearance (colour, shine, whey presence, visual smoothness, adhesiveness, teaspoon filling, consistency uniformity), seven described the texture in the mouth (smoothness, melting, creaminess, thickness, firmness, yield stress, fat film), four described the taste (sweet, sour, bitter and astringent), five described the flavour (yoghurt, milky, quark, peach, nectar) and the characteristics described the body of the product and its overall quality. The intensity of the sensory attributes was rated by experts on a 10-cm unstructured linear scale with edge terms ranging from “none” to “very intense”. The smell of the sample was evaluated first by slightly tilting the lid, then the visual aspect of the sample’s appearance was evaluated after opening the container. The next aspects to be evaluated were the texture aspects perceived by mouth, by dipping a teaspoon and by tasting the sample. Finally, the taste sensations perceived in the mouth were analysed. A sensory profiling assessment was performed by a team of 10 trained experts (ISO standard 8586:2012 [46]) (age 35–52) with experience in the evaluation of yoghurts.

*The Semi-consumer Test Method:* In order to assess the acceptability of the developed yoghurts, the acceptability of their appearance, odour, consistency, taste and overall acceptability (based on associations related to appearance, odour, consistency and taste of the samples evaluated) were evaluated. A hedonic assessment was carried out using a 9-point scale with ranges from “extremely dislike” to “extremely like” and, in the case of purchase intentions, from “definitely will not buy” to “definitely will buy” [47,48,49]. For the semi-consumer evaluation [20], 30 students from the Department of Human Nutrition (aged 19–31 years) who regularly consume yoghurt and have no milk allergies were randomly recruited based on their willingness and interest to participate in the study.

*Testing Conditions:* The expert test and the semi-consumer test were performed in an accredited sensory laboratory (accreditation number AB 564) that complies with the requirements of ISO standard 8589:2007 [50]. The yoghurts were evaluated by experts and consumers in individual test booths with controlled lighting, temperature and humidity to ensure that the evaluators could properly focus on the product samples being evaluated. Each booth was equipped with an ANALSENS computer system (Cogitos, Sopot, Poland) which allowed the yoghurt to be assessed and data to be collected. The yoghurt assessments were carried out in the morning and early afternoon (in the case of an expert assessment, there were two sessions in one day with a 3 h break).

*Sample Preparation and Presentation:* The yoghurt samples for the expert and consumer evaluations were prepared in cylindrical containers (ø, 50 mm; height, 50 mm; volume, 100 mL). The containers were coded with 3-digit codes, then placed randomly on a tray and served for evaluation at 7 °C. All six of the yoghurt samples were evaluated one by one directly from the test containers. The assessors used still mineral water as a neutraliser between the tested samples.

#### 2.2.2. Instrumental Analysis

##### Textural Properties

The texture parameters, firmness (N) and adhesiveness (Ns), of the yoghurts were analysed using a texture analyser (TA.XT Plus, Stable Micro Mixtures, Surrey, UK) with a 5 kg load cell at 20 °C, and a 0.5-cm diameter cylindrical flat probe (P/0.5R). The measurement parameters were speed, 1.0 mm/s; trigger force, 1 g, and penetration depth, 5 mm. Samples were measured in cylindrical containers (ø, 50 mm; height, 50 mm; volume, 100 mL). The experiment was conducted in three replications. The data were analysed using the Exponent v6.1.4.0 equipment software (Stable Micro Mixtures, Surrey, UK) [51,52].

##### Yield Stress

The yield stress of the yoghurts was measured using a rheometer (DV3T, Brookfield, Middleboro, MA, USA). The measurement’s parameters were temperature, 20 °C; vane spindle V74 with a torque range HA; and a shear rate from 0.01–100 s^−1^. Samples were measured in cylindrical containers (ø, 50 mm; height, 50 mm; volume, 100 mL). The experiment was conducted in three replications. The data were analysed using the software provided with the rheometer [51,53].

##### Physical Stability—CSA Method

The yoghurt stability was investigated using space- and time-resolved extinction profile (STEP) technology. This technique uses gravitational fields to accelerate the occurrence of instability phenomena such as sedimentation, flocculation or creaming [53]. The physical stability of the yoghurts was determined using an analytical centrifuge LUMiSizer 6120-75 (L.U.M. GmbH, Berlin, Germany) by measuring the intensity of transmitted near-infrared light in suspension and recording the light intensity profiles as a function of time and the position of the sample (“fingerprints”) [54,55]. The measurement parameters were wavelength, 870 nm; volume, 1.8 mL of dispersion; light factor, 1; 4000 rpm; experiment time, 50 min; interval time, 10 s; and temperature, 25 °C. The experiment was conducted in three replications. The data were analysed using the delivered software (SepView 6.0; LUM, Berlin, Germany) and the instability index was calculated [55].

##### Colour Parameters

Colour components (L *, a * and b *) were determined with the usage of a Minolta CR-200 colorimeter (Minolta, Osaka, Japan). The measurement parameters were a D65 light source and, a measuring head hole of 8 mm. Colour parameters were analysed using the CIEL * a * b * system. The measurements were made in the reflectance. The total colour difference (ΔE) parameter was also calculated to determine the differences in colour between the control yoghurt (C) and the yoghurt with an inulin addition (C1) or the oolong tea-infused yoghurt (O), separately. The differences between the oolong tea-infused yoghurt (O) and the oolong tea-infused yoghurt with inulin (O1, O2, O3) were calculated [56] as follows:

(1) $$\Delta E=\sqrt{{\left(L_{c}^{*}-L_{T}^{*}\right)}^{2}+{\left(a_{c}^{*}-a_{T}^{*}\right)}^{2}+{\left(b_{c}^{*}-b_{T}^{*}\right)}^{2}}$$

where $L_{c}^{*},{\;a}_{c}^{*},b_{c}^{*}$ refers to the colour parameters of the C or O yoghurts and $L_{T}^{*},{\;a}_{T}^{*},b_{T}^{*}$ refers to the colour parameters of the oolong tea-infused yoghurts or the yoghurts with an inulin addition, respectively.

#### 2.2.3. Statistical Analysis

Statistical analysis of the results was performed using Statistica 13.3 software (TIBCO Software Inc. Palo Alto, CA, USA). A one-way analysis of variance (ANOVA) with the NIR Fisher post hoc test at a significance level *p* ≤ 0.05 was used to determine the differences in the intensiveness of the different sensory characteristics and Tukey’s test (at a significance level α = 0.05) for instrumental analysis. In addition, a Principal Component Analysis (PCA) was performed using ANALSENS NT to determine the differences between the samples and the correlations of individual parameters.

## 3. Results and Discussion

### 3.1. Sensory Analysis

#### 3.1.1. Quantitative Descriptive Profile Analysis of Oolong Tea-Infused Yoghurts with Inulin in Comparison to Plain Yoghurt (Expert Test)

The use of an oolong tea addition to yoghurt influenced changes in the sensory quality profile of yoghurt. The yoghurt profile also changed due to the addition of inulin (Supplementary Material-Table S2).

**Figure 1:**
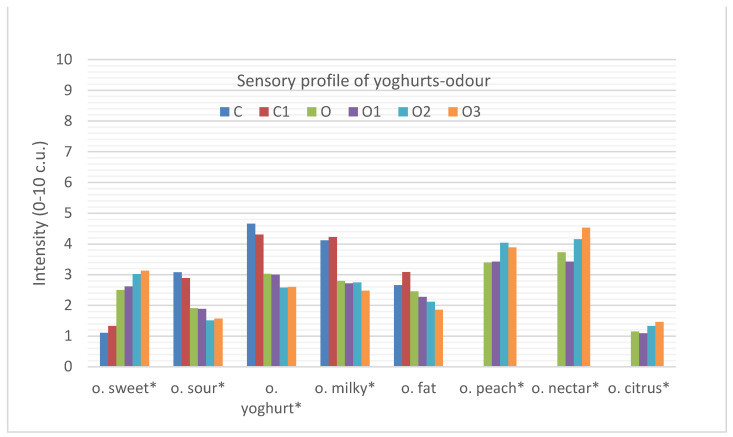
Sensory quality profile of yoghurts describing odour attributes. C, control; C1, control with 6% inulin; O, oolong tea; O1, oolong tea with 3% inulin; O2, oolong tea with 6% inulin; O3, oolong tea with 9% inulin (* *p* ≤ 0.05 differ significantly).

Figure 1 shows the changes in the sensory quality profile of yoghurt odours due to the infusion of oolong tea as well as the addition of inulin to the different yoghurt types.

The natural yoghurt (C) was characterised by an intense milky (4.1 c.u. (contractual units)) and yoghurt odour (4.7 c.u.), as well as a sour (3.1 c.u.) and fatty odour (2.7 c.u.). However, the use of oolong tea (O) in the production of yoghurt significantly reduced the perception of sour (1.9 c.u.), milky (2.8 c.u.) and yoghurt (3.0 c.u.) odours and increased the perception of sweet odours (2.5 c.u.). In addition, positive intensive fragrance notes of peach (3.4 c.u.), nectar (3.7 c.u.) and citrus (1.2 c.u.), characteristic of the applied oolong tea, appeared, which significantly differentiated it from the plain yoghurt. The same relationships were found in another study [20].

The addition of inulin to yoghurt did not significantly affect the changes in odour of both the natural (C and C1) and the oolong tea-infused yoghurts (O, O1, O2, O3). However, a trend was observed, i.e., the control tea-infused yoghurt (O) and tea-infused yoghurts with inulin did not differ in terms of odour, but they differed significantly from the plain yoghurt (C) and plain yoghurt with inulin (C1), but this was mainly due to the addition of tea.

**Figure 2:**
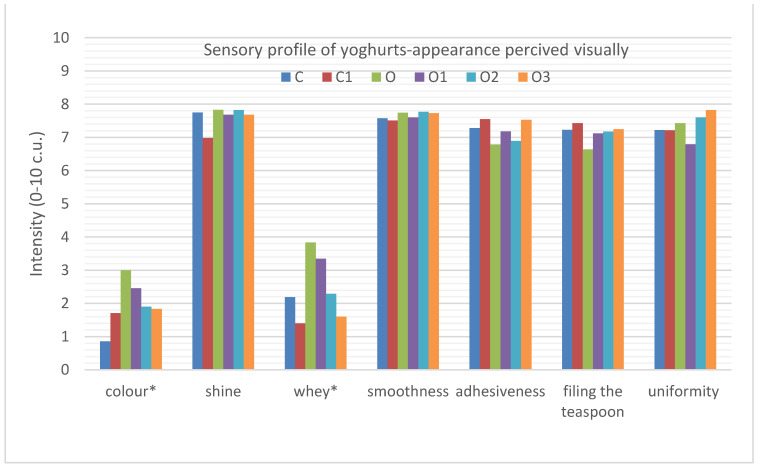
Sensory quality profile of yoghurts describing the appearance attributes perceived visually. C, control; C1, control with 6% inulin; O, oolong tea; O1, oolong tea with 3% inulin; O2, oolong tea with 6% inulin; O3, oolong tea with 9% inulin (* *p* ≤ 0.05 differ significantly).

When analysing the appearance characteristics of the yoghurt, it can be seen that the only statistical differences between the samples were in the presence of whey run-off in the samples and the colour intensity (Figure 2).

The whey presence in all of the samples was low, but it was significantly lower in the plain yoghurt (2.2. c.u.) than in the sample with oolong tea (3.8 c.u.). A similar relationship was found in a study conducted by Świąder et al. [20], where the control yoghurt sample (plain yoghurt) had a significantly lower whey presence (1.9 c.u.) than the oolong tea yoghurt sample (4.9 c.u.). In addition, it could be observed that the incorporation of inulin at the level of 6 and 9% significantly reduced the presence of whey in the yoghurts with oolong tea (O, 3.8 c.u.; O2, 2.3 c.u.; O3, 1.6 c.u.). Although the presence of whey was also reduced in the sample of natural yoghurt with inulin (1.4 c.u.) compared to the sample of natural yoghurt (2.2 c.u.), they did not differ significantly statistically.

The analysed samples differed significantly statistically in colour intensity. The addition of oolong tea to natural yoghurt significantly changed the colour from light white to slightly dark creamy (C, 0.9 c.u.; O, 3.0 c.u., respectively). A similar relationship was observed in a previous study [20], where the control sample of plain yoghurt had a bright white colour of 1.0 c.u. while the oolong tea-infused yoghurt was significantly darker at 3.2 c.u. The addition of inulin to the oolong tea-infused yoghurt had a significant effect on the lightening of the colour (O, 3.0 c.u.; O1, 2.5 c.u.; O2, 1.9 c.u.; O3, 1.8 c.u.), in comparison to the colour of natural, plain yoghurt with inulin (1.7 c.u.).

All of the analysed yoghurts were characterised by an intensive gloss on the yoghurt’s surface (7–7.8 c.u.) and a high smoothness assessed visually (7.5–7.8 c.u.), and also by uniform consistency (6.8–7.8 c.u.). They were also characterised by high adhesiveness (6.8–7.5 c.u.) as well as by forming convex meniscuses on the teaspoon (6.6–7.4 c.u.).

**Figure 3:**
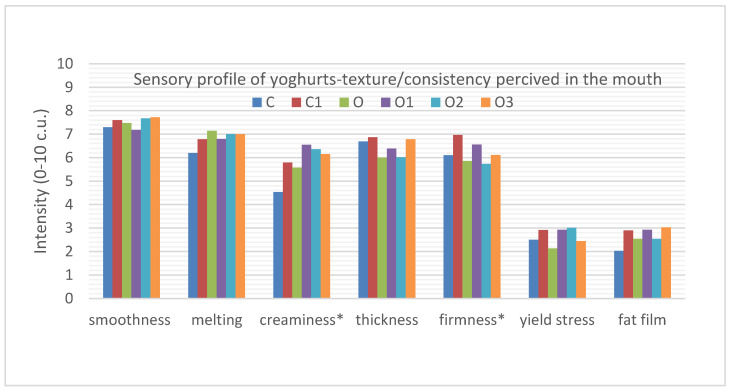
Sensory quality profile of yoghurts describing the texture/consistency attributes perceived in the mouth. C, control; C1, control with 6% inulin; O, oolong tea; O1, oolong tea with 3% inulin; O2, oolong tea with 6% inulin; O3, oolong tea with 9% inulin (* *p* ≤ 0.05 differ significantly).

When analysing the texture and consistency characteristics of the evaluated oral samples, it can be noted that they differed significantly statistically only in firmness and creaminess (Figure 3).

The addition of oolong tea to yoghurt did not affect the firmness in comparison to natural, plain yoghurt (O, 5.9 c.u.; C, 6.1 c.u., respectively). In studies conducted on tea-infused yoghurts [20], there was also no significant difference observed between the samples of plain yoghurt (5.3 c.u.) and oolong tea-infused yoghurt (5.8 c.u.). However, differences in firmness were noticeable when inulin was added to the natural yoghurt. The addition of inulin to the natural yoghurt significantly increased the firmness of the natural yoghurt from 6.1 to 7.0 c.u. No such significant statistical difference in firmness was found when inulin was added to oolong tea-infused yoghurt.

The addition of oolong tea as well as inulin improved the creaminess of the yoghurts. The highest difference in creaminess was observed between the natural yoghurt (C, 4.5 c.u.) and oolong tea-infused yoghurts with the addition of 3, 6 and 9% inulin (O1, 6.6 c.u.; O2, 6.4 c.u.; O3, 6.2 c.u.). All of the analysed samples had a high thickness in the mouth (6–6.9 c.u.), the highest was detected in natural yoghurt with inulin and with oolong tea and inulin 9%, but these differences were not statistically significant.

Yoghurts were also characterised by a high dispersibility in the mouth, but no statistically significant differences were found between the analysed samples (6.2–7.1 c.u.). The same was true for the perceived smoothness in the mouth. The yoghurt samples were characterised by high smoothness, but there were no statistically significant differences between them (7.2–7.7 c.u.).

The added yoghurt samples were characterised by low yield stress (2.1–3.0 c.u.) and had a slightly perceptible fatty film (2–3.0 c.u.). A fat film was most noticeable in the samples of natural yoghurt with inulin and oolong yoghurt with inulin of a 3 and 9% concentration. The highest viscosity (yield stress) was found in natural yoghurt with inulin and in the yoghurts with oolong tea and inulin concentrations of 3 and 6%. There was no statistically significant difference between the samples for these two characteristics. In another study [20], there were no statistically significant differences in the texture in the mouth between samples of natural yoghurt and oolong tea-infused yoghurt.

**Figure 4:**
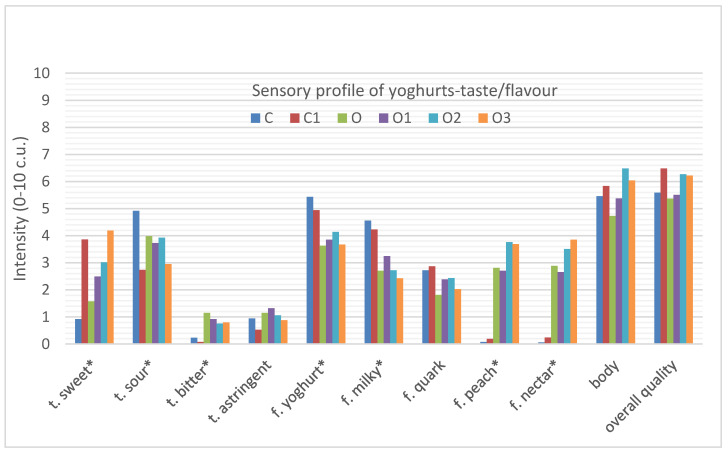
Sensory quality profile of yoghurts describing the taste (t) and flavour (f) attributes as well as body and overall quality. C, control; C1, control with 6% inulin; O, oolong tea; O1, oolong tea with 3% inulin; O2, oolong tea with 6% inulin; O3, oolong tea with 9% inulin (* *p* ≤ 0.05 differ significantly).

In the case of taste profile, the addition of oolong tea to natural yoghurt caused changes in perceived taste/flavour qualities (Figure 4).

Sour, milky and yoghurt flavours were most noticeable in natural, plain yoghurt, while sweet and bitter tastes, and green tea, peach and nectar flavours were most noticeable in the yoghurt with oolong tea. The sweet (O, 1.6 c.u.) and bitter taste (O, 1.5 c.u.), as well as peach (O, 2.8 c.u.) and nectar flavours (O, 2.9 c.u.) found in the yoghurt with oolong tea significantly differentiated this type of yoghurt from natural yoghurt. The addition of inulin to natural yoghurt significantly increased the sweet taste. A similar relationship was observed in the case of the yoghurt with oolong tea, where the addition of inulin increased the intensity of the sweet taste and the sweetness increased proportionally to the increase in the concentration of inulin used. Oolong tea-infused yoghurt was characterised by a less noticeable sour taste and milky, yoghurt flavour.

All of the tested yoghurts were harmonised in terms of the intensity of all the positive attributes. The samples of plain yoghurt did not differ significantly from the oolong tea yoghurt in terms of product body (C, 5.5 c.u.; O, 4.7 c.u. respectively), which corresponds to an earlier study conducted by Świąder et al. [20].

Similarly, the addition of inulin to yoghurts had no statistically significant effect on the body of plain yoghurt or oolong tea yoghurt, although an increase in the figures for the control yoghurts with inulin (C, 5.5 c.u., C1, 5.8 c.u.) and the yoghurts with oolong tea and inulin (O, 4.7 c.u.; O1, 5.4 c.u.; O2, 6.5 c.u.; O3, 6.0 c.u.) can be seen.

All of the samples were characterised by a fairly high overall quality. The plain yoghurt did not differ from the oolong tea yoghurt in terms of overall quality (C, 5.6 c.u.; O, 5.4 c.u., respectively). The same relationship was noted in another study [20], where samples of plain yoghurt and oolong tea-infused yoghurt did not differ statistically between each other (plain yoghurt, 5.9 c.u.; oolong tea yoghurt, 5.6 c.u.).

Although the addition of inulin increased the overall quality of natural yoghurt from 5.5 c.u. to 6.5 c.u., it was not a statistically significant difference. In the case of yoghurt with oolong tea, this change was most pronounced with the addition of 3% (6.3 c.u.) inulin and 6% (6.2 c.u.) inulin, but these differences were also not statistically significant.

In the literature, data on the quality of yoghurts with tea show that their sensory quality was evaluated using hedonic scales, which only allowed for the assessment of the acceptability and enjoyment of yoghurts with tea, but they did not include the results of sensory quality evaluations carried out by experts, which would provide detailed information about the quality profile of the evaluated products and give information on which of the yoghurt characteristics affected their acceptability, or lack thereof [41,42,43,44]. The only study aimed at determining the quality of yoghurts with tea by conducting detailed expert studies and verifying them with consumer studies to which we could refer in our study was carried out by Świąder et al. [20]. Additionally, no study was found that evaluated the sensory quality of yoghurts infused with semi-fermented oolong tea, nor was a study on the addition of inulin to this type of tea-infused yoghurt carried out by a panel of experts.

There are studies that support the beneficial effects of catechin-rich green tea and inulin on body composition in overweight adults, based on the consumption of a green tea beverage with inulin [57], but these focus on the health aspect of the beverage and not on its sensory quality and acceptability to the participants.

According to the obtained results, the addition of oolong tea to yoghurt resulted in positive changes in the sensory evaluated perception of peach and nectar odours and flavours. Unfortunately, the tea infusion also resulted in negative changes, the most unpleasant of which was the presence of a bitter taste, also those yoghurts were characterised by the whey presence and a darker intensity of colour. Those negative features were balanced with the addition of inulin. Inulin is well known for its texture-enhancing properties in food products [29,30,31]. Inulin, in addition to its effect on texture changes, is also used as a fat or sugar substitute in the form of a low-calorie sweetener [32] to improve the organoleptic quality of products [29] and as a nondigestible fibre to form gels and increase the viscosity of products [31,58]. Inulin can be used in dairy and non-dairy products, but we see the most applications of inulin in the dairy industry, where is used as a prebiotic and as a sugar and fat replacer [31,58]. The use of inulin makes it possible to produce a reduced- or low-fat, texturized, symbiotic cheese [29], or a low-fat dietary yoghurt with improved textural and sensory properties comparable to a higher-fat yoghurt [30,31].

Based on the research carried out with inulin and oligofructose, it can be concluded that the influence of inulin on the sensory properties of products depends on many factors, i.e., the type of inulin, its degree of polymerisation [35,36,37,38], its concentration (1–6% yoghurts [35,38,39,40]), the product in which it is used (dairy [38,39,40], bakery products [59,60]), the role it has to play in a product (replacing fat and sugar or improving organoleptic properties and texture), fat or sugar reduction (cake, 50–100% fat reduction [59]; cake, 30% sugar reduction [60]), and the type of substances added and their quality (whole milk [36], skimmed milk [35]).

As far as I know, there are no studies that have assessed the effect of inulin on the textural and sensory properties of oolong tea-infused yoghurt.

In our study, as part of flavour improvements in oolong tea yoghurts, the addition of inulin improved the yoghurt creaminess for both oolong tea-infused yoghurt and natural, plain yoghurt, and the firmness of natural yoghurt. The addition of inulin also significantly improved the sweet odour and taste of the oolong tea-infused yoghurt and the plain yoghurt. However, these changes did not affect the overall quality of the yoghurts as assessed by experts, and the acceptability and willingness to buy as assessed by consumers.

#### 3.1.2. Principal Component Analysis (PCA)

**Figure 5:**
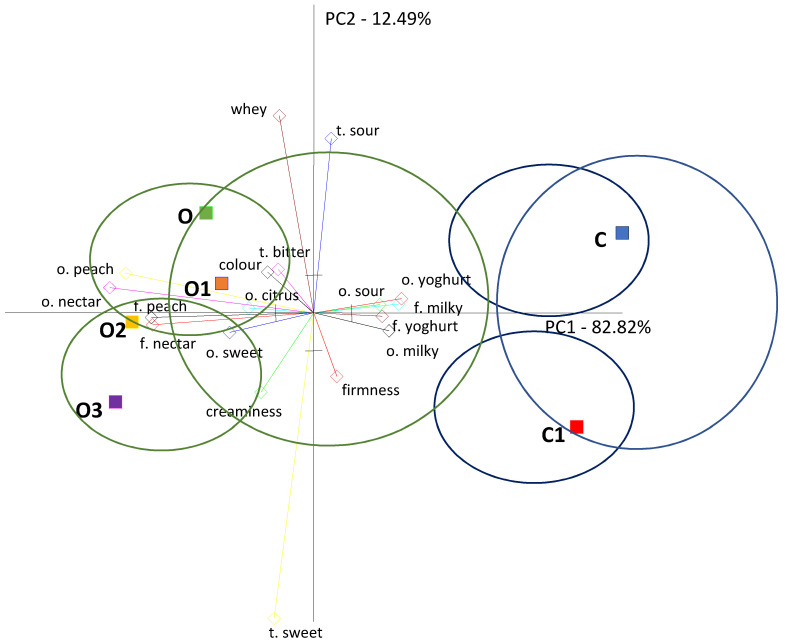
Principal Component Analysis (PCA) of the following yoghurt samples: C, control; C1, control with 6% inulin; O, oolong tea; O1, oolong tea with 3% inulin; O2, oolong tea with 6% inulin; O3, oolong tea with 9% inulin.

A principal component analysis assessment was carried out for all of the evaluated yoghurts. In order to make the relations between the samples and the factors defining their quality more visible, of the 33 attributes only those that significantly statistically (*p* ≤ 0.05) differentiated the evaluated samples were selected for the PCA presentation (Figure 5).

A principal component analysis of the results of six evaluated yoghurt samples showed that sample variation was attributed to the first main component (PC1), which accounted for 82.82% of the total variability and was related to the peach and nectar odour and flavour that differentiated the analysed samples of yoghurts (Figure 5). The second component (PC2) constituted 12.49% of the general variable and was related to sweet taste, sour taste and the presence of whey.

From the PCA image and the location of the samples, it can be seen that the yoghurt samples differed in quality. In addition, they formed two distinct clusters, among which two additional clusters could be identified. The first cluster contained samples of natural yoghurt, while the second cluster contained oolong tea-infused yoghurts. It can be seen that oolong tea-infused yoghurts with inulin and without formed two additional clusters, in which the oolong tea-infused yoghurt sample with inulin 3% was similar to the control sample with oolong tea only, whereas in the second cluster there were samples of oolong tea-infused yoghurts with a higher inulin content, i.e., 6% and 9%.

The yoghurt samples with oolong tea were characterised by an intense peach and nectar aroma and flavour. The peach and nectar flavours were most noticeable in the oolong tea samples with inulin at 6 and 9% concentrations, as reflected in the Quantitative Descriptive Profile analysis (QDP). In addition, the variation of samples in terms of sweet taste and smell is noticeable in the PCA graph and in the QDP results. The sweetness was more pronounced in the oolong tea yoghurt than in the plain yoghurt, and the addition of inulin influenced the intensity of the sweet taste. It can be seen that the control sample with inulin was sweeter than the control sample without inulin (plain yoghurt), which was definitely more acidic. The sweetness of the oolong tea samples increased with the addition of inulin.

The oolong tea-infused yoghurts were also creamier, and the addition of inulin improved the creaminess of the yoghurts for both the oolong tea-infused yoghurt and the natural, plain yoghurt, which was also reflected in the QDP analysis.

The oolong tea-infused yoghurts also had a bitter taste and higher whey flow, the highest in the oolong tea-infused yoghurt sample and the lowest in the samples on the other side of the PCA graph, i.e., the control sample (plain yoghurt) with inulin and the oolong tea-infused yoghurts with 6 and 9% inulin, which also corresponds to the results of the QDP analysis. The whey presence in both the natural and oolong tea yoghurts was reduced by the addition of inulin, which also corresponds to the results of the QDP analysis.

The natural yoghurt samples were concentrated on the opposite side of the PCA graph, showing different defining characteristics. Natural yoghurts were correlated with yoghurt and milk taste and smell. Additionally, natural yoghurt had the most intense acid taste and smell. In the natural yoghurt cluster, however, the sample of natural yoghurt with inulin was more compact and sweeter, and thus less acidic than the natural yoghurt forming a separate cluster. These differences were also reflected in the Quantitative Descriptive Profile analysis.

A study of the sensory quality of yoghurts infused with black, green and oolong tea showed a similar correlation, where oolong tea had an intense peach flavour and aroma that positively correlated with overall yoghurt quality, unlike other yoghurts with black and green tea, while the control samples of natural yoghurt were characterised by a milky, yoghurt-like and sour taste and smell [20].

#### 3.1.3. Acceptability of Oolong Tea-Infused Yoghurts with Inulin and Willingness to Buy Evaluated by Consumers (Semi-Consumer Evaluation)

In the addition to sensory testing of the yoghurts by experts, a semi-consumer evaluation was carried out with 30 consumers who regularly consume yoghurt. The purpose of the evaluation was to verify how consumers react to the developed yoghurts, whether they accept them and whether they are willing to buy them. Consumer evaluation is as important in designing a new product as an expert evaluation of its quality.

**Figure 6:**
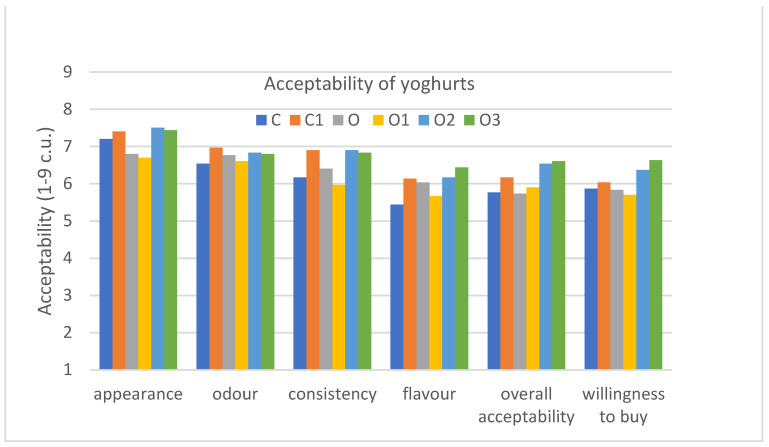
Graph of acceptability of the following six analysed yoghurts: C, control; C1, control with 6% inulin; O, oolong tea; O1, oolong tea with 3% inulin; O2, oolong tea with 6% inulin; O3, oolong tea with 9% inulin (the samples were not statistically different at *p* ≤ 0.05).

In the semi-consumer evaluation, the consumers were asked to rate the acceptability of the appearance, smell, texture, taste and the overall acceptability, as well as willingness to buy the evaluated products, which differed in the addition of inulin and were compared to a control natural yoghurt (plain) and oolong tea without inulin on a 9-point hedonic scale (Supplementary Material-Table S3) (Figure 6).

The results obtained in the semi-consumer test indicate that the differences between the samples were not significant, but trends can be observed that are worth describing and comparing with the expert studies carried out using the QDP method. The yoghurt with oolong tea and inulin (6% and 9%) as well as the natural yoghurt with inulin (6%) were characterised by the highest appearance acceptability in comparison to the yoghurt with oolong tea (O2, 7.5 c.u.; O3, 7.4 c.u.; C1, 7.4 c.u.; O, 6.8 c.u.; but the difference was not statistically significant), which may result from the fact that these yoghurts, due to the addition of inulin, according to the QDP evaluation, were characterised by a lower whey presence. Additionally, according to the QDP study, the addition of inulin to oolong tea yoghurt, which was characterised by a darker creamy colour compared to natural yoghurt, improved the colour of the yoghurt from dark creamy to light white.

In the case of the odour acceptability of yoghurts, it can be observed that natural yoghurt with inulin (C1, 7.0 c.u.), as compared to natural yoghurt (C, 6.5 c.u.), was characterised by a higher acceptability, but the difference was not statistically significant. On the basis of the QDP results, it may be due to the addition of inulin, which influenced the more intensive perception of sweet smell. In addition, the yoghurt with oolong tea (O, 6.8 c.u.) had a higher odour acceptability than natural yoghurt (C, 6.5 c.u.), which, on the basis of the QDA analysis, could be due to the occurrence of a pleasant nectar, peach and sweet smell resulting from the presence of oolong tea and additionally enhanced by inulin. However, the statistical analysis showed no statistically significant differences between the samples in terms of the analysed odour acceptability.

When evaluating the acceptability of the consistency of the evaluated yoghurts, it can be seen that the samples with the addition of inulin at the levels of 6 and 9% were characterized by higher acceptability, both in the case of the natural yoghurt (C1, 6.9 c.u.) and the yoghurts with oolong tea (O2, 6.9 c.u.; O3, 6.8 c.u.) compared to the control samples (C, 6.2 c.u.) and the oolong tea yoghurt (O, 6.4 c.u.), while in the statistical evaluation there were no statistically significant differences between them in this evaluated attribute.

The results of the evaluation of taste acceptability by consumers show that they accepted the taste of oolong tea-infused yoghurt (O, 6.0 c.u.) more than that of natural yoghurt (C, 5.4 c.u.). In addition, the yoghurt samples with inulin were more acceptable (C1, 6.1 c.u.; O2, 6.2 c.u.; O3, 6.4 c.u.). However, looking at the results of the statistical evaluation, it can be seen that the samples were also not significantly different in taste acceptability. On the basis of the QDP evaluation, it can be concluded that this could be due to the fact that the oolong tea yoghurt samples had a very intense and pleasant peach and nectar taste as well as a sweet taste, which was further intensified by the addition of inulin.

Taking into account the acceptability of all the evaluated attributes, it can be seen that the evaluators assessed the oolong tea-infused yoghurt with an inulin content of 6 and 9% (O, 6.5 c.u.; O3, 6.6 c.u.) and the natural yoghurt with inulin (C1, 6.2 c.u.) the highest. However, there was no statistically significant difference between the samples in terms of their overall acceptability.

The consumers were also asked about their willingness to buy the yoghurt samples evaluated, and the yoghurts they were most willing to buy were the oolong tea-infused yoghurt with an addition of 6% (O2, 6.4 c.u.) and 9% inulin (O3, 6.6 c.u.), while the statistical evaluation showed no differences in willingness to buy between the evaluated samples.

In a study conducted to evaluate the sensory quality of yoghurts infused with different teas, yoghurts with oolong tea and natural yoghurts without any additives were found to be the most acceptable, significantly more than those with green or black tea. At the same time, yoghurt with oolong tea and natural yoghurt did not differ significantly statistically in terms of acceptability [20], which is in line with our study. The same trend was found in the purchase intention. Consumers were most likely to buy the oolong tea yoghurt and the control yoghurt, which did not differ significantly in terms of purchase intention [20], which is in accordance with our study.

### 3.2. Instrumental Analysis

#### 3.2.1. Textural Properties of Functional Oolong Tea-Infused Set Type Yoghurt with Inulin

A set type yoghurt’s texture is semisolid due to presence of milk proteins that have an ability to form a three-dimensional network. Thanks to the fermentation process, the high net negative charge on the casein micelles is being reduced, which is consequently responsible for milk gelation. Casein micelles and denatured whey proteins aggregate by forming hydrophobic and electrostatic bonds [61]. These gel structures, however, might be influenced by several factors, such as, among others, tea [62] and inulin addition [61]. As it was described previously, the oolong tea infusion resulted in a deterioration of the texture parameters of set yoghurts [20]. In the conducted experiment, the oolong tea influence was counterbalanced by an addition of inulin. The results of the texture parameters are shown in Table 1.

It was found that an inulin addition in a concentration of 3% resulted in increasing firmness, from 0.706 N for the infused-tea yoghurts to 0.995 N for the yoghurts with oolong tea and inulin. This might be due to ability of inulin to incorporate into the yoghurt matrix, conferring and enforcing the existent bonds between milk proteins [63]. However, after increasing the inulin concentration to 6%, a deterioration in the yoghurt texture was observed, with the firmness lowered to 0.728 N. This might be related to the inulin dispersed among the casein micelles, consequently interfering with the protein matrix’s formation [64]. On the other hand, the highest tested inulin concentration (9%) resulted in the strengthening of the structure, as firmness was increased to 1.032 N. The firmness of yoghurt is directly dependent on its total solids [65], which is why a higher inulin concentration might cause better conditions for the protein matrix’s formation. In addition, inulin itself can form a gel structure, especially in higher concentrations, and stabilize the gel structure by forming polysaccharide-protein hydrogels [66]. The obtained firmness results were reflected in the sensory evaluation. A higher (9%) inulin concentration in oolong tea-infused yoghurts also determined the adhesiveness of the yoghurt, which, along with firmness, is very important factor, especially for set type yoghurts. The addition of 9% inulin, in comparison to other samples, increased the yoghurt’s adhesiveness by more than 1.6×. Thanks to a higher adhesiveness, the oolong tea-infused yoghurts with 9% inulin were also charactered by higher scores for sensory evaluated adhesiveness and thickness in the mouth.

#### 3.2.2. Yield Stress of Functional Oolong Tea-Infused Set Type Yoghurt with Inulin

Yield stress causes the initial resistance of yoghurt to flow under stress, and depends on the interactions between the casein, whey proteins and inulin. For the tested yoghurts, it was reported that yield stress (Table 1) was reduced (from 147.4 to 121.4 Pa) in the oolong tea infusion, whereas an inulin addition improved the yield stress; however, the differences were not statistically significant. The deterioration in the yield stress of set yoghurt infused with tea was tested previously [20,67] and might be caused by interactions of polyphenolic compounds with proteins present in yoghurt [68].

#### 3.2.3. Physical Stability—CSA Method of Functional Oolong Tea-Infused Set Type Yoghurt with Inulin

**Figure 7:**
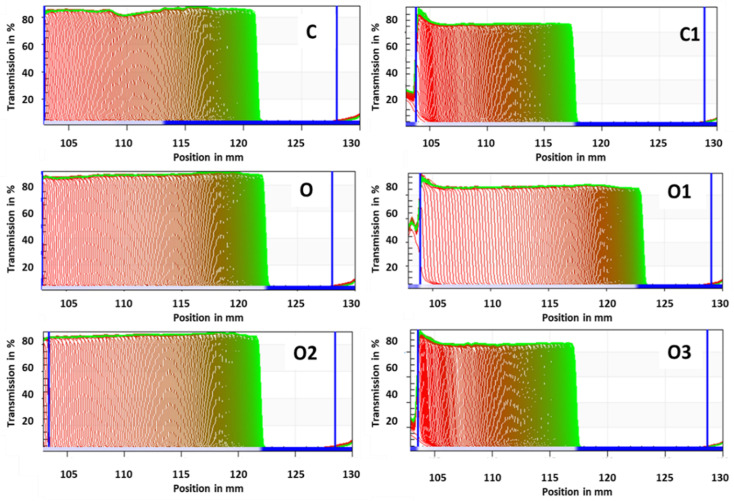
Influence of different tea infusions on the yoghurt’s transmission profiles presented, enabling LUMi Sizer^®^ analysis. C, control; C1, control with 6% inulin; O, oolong tea; O1, oolong tea with 3% inulin; O2, oolong tea with 6% inulin; O3, oolong tea with 9% inulin.

Stability is a very important factor in set type yoghurt because syneresis, serum release from its gel structure, is regarded by consumers as a technological defect. That is why the characteristic of yoghurt stability is very important [67]. The yoghurts’ stability was examined with a multi-sample analytical centrifuge using STEP (space-time resolved extinction profiles) technology. The transmission profiles are presented in Figure 7.

By analysing the evolution of the light transmission profiles (“fingerprints”), it can be stated that the sedimentation phenomenon occurred in each sample. The migration of particles to the bottom of the probe (right side of the graph, Figure 7) was observed, which consequently led to the clarification of the upper parts of the sample, with water separation, and thus a higher intensity of transmitted light was noted.

None of the tested yoghurts, regardless oolong tea or inulin addition, were stable during the time of stability determination. The release of serum (syneresis) was observed. The separation is related to the instability of a yoghurt’s structure, especially the casein and whey protein aggregates that build the gel network [61]. A slightly lower stability, which was also visible in the sensory measured whey presence, was observed in the yoghurts with an inulin addition.

**Figure 8:**
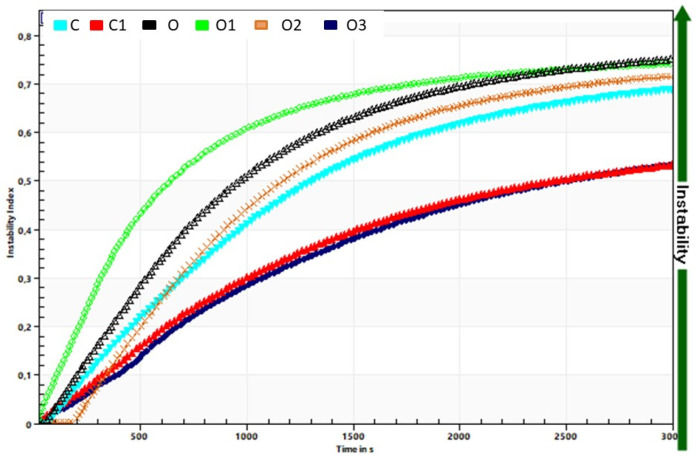
Influence of tea extract addition on the stability of yoghurt in comparison to plain yoghurt. C, control; C1, control with 6% inulin; O, oolong tea; O1, oolong tea with 3% inulin; O2, oolong tea with 6% inulin; O3, oolong tea with 9% inulin.

The instability index (Table 1, Figure 8) was lower in the case of inulin addition in oolong infused-tea yoghurts, which indicates that the gel structure might be stronger than that created only with ingredients originating from native milk. The authors of [37] also found that the increased use of inulin negatively affected some physical properties, e.g., whey separation, consistency and organoleptic scores.

#### 3.2.4. Colour Parameters of Functional Oolong Tea-Infused Set Type Yoghurt with Inulin

The QDP analysis revealed that the tested oolong tea yoghurts had an intense colour, but after an inulin addition these scores were reduced. This means that inulin influences the perception of the intensity of a yoghurt’s colour.

In instrumental colour measurement, the samples containing oolong tea showed lower L * values in comparison to control products (the loss of lightness was observed), the lightness values shifted after adding 3% inulin and dropped with a higher amount of inulin (Table 2). Lower L * values after an inulin addition to yoghurt was also observed by the authors of [61]. After analysing the ΔE parameter, it can be said that changes in the colour caused by an inulin addition are very small and are visible only to a very experienced observer, but differences between the samples with and without oolong tea are visible even to unexperienced observers, as indicated by a ΔE greater than two.

## 4. Conclusions

The results of the conducted study indicate an appropriate combination of yoghurt cultures, oolong tea and inulin in the creation of functional products, characterised by a high sensory quality and acceptability, as well as health-promoting properties resulting from the raw ingredients used. The use of oolong tea, which is rich in catechins and imparts to yoghurt a natural peach–nectarine flavour without the need for synthetic additives, brings additional value to natural yoghurt. The use of inulin, on the other hand, increased the sweetness of the yoghurt and improved the appearance and texture of the yoghurt, which was impaired by the use of oolong tea in yoghurt production. Thanks to the presence of inulin in such a large amount, it would be possible to add the health claim that, due to the level of insulin in this product, the consumption of this yoghurt will contribute to normal bowel function by increasing stool frequency, as well as the nutritional claim that the yoghurt is high in fibre. Both the sensory and instrumental analysis confirmed the possibility of using these ingredients in set type yoghurts; however, research into the health and nutritional aspects of these products should be carried out in the future.

## Figures and Tables

**Table 1:** Physical properties of yoghurts. C, control; C1, control with 6% inulin; O, oolong tea; O1, oolong tea with 3% inulin; O2, oolong tea with 6% inulin; O3, oolong tea with 9% inulin.

Sample	Yield Stress	Texture		Instability Index
	(Pa)	Firmness (N)	Adhesiveness (Ns)	
C	147.4 ^b^ ± 9.9	0.895 ^ab^ ± 0.064	−0.055 ^b^ ± 0.000	0.689 ^b^ ± 0.010
C1	144.3 ^ab^ ± 8.6	1.050 ^b^ ± 0.089	−0.075 ^b^ ± 0.008	0.532 ^a^ ± 0.008
O	121.4 ^a^ ± 6.0	0.706 ^a^ ± 0.072	−0.068 ^b^ ± 0.006	0.752 ^d^ ± 0.031
O1	140.0 ^ab^ ± 2.9	0.995 ^b^ ± 0.076	−0.067 ^b^ ± 0.010	0.740 ^d^ ± 0.27
O2	121.8 ^a^ ± 8.2	0.728 ^a^ ± 0.094	−0.073 ^b^ ± 0.013	0.715 ^c^ ± 0.34
O3	140.3 ^ab^ ± 15.9	1.032 ^b^ ± 0.096	−0.120 ^a^ ± 0.035	0.531 ^a^ ± 0.011

Values are mean ± SD (*n* = 3), ^a, b, c, d^ Values followed by the same letter within a column do not differ significantly according to Tukey’s test (*p* < 0.05).

**Table 2:** Colour parameters and the total colour difference parameter in yoghurts obtained without or with the addition of tea. C, control; C1, control with 6% inulin; O, oolong tea; O1, oolong tea with 3% inulin; O2, oolong tea with 6% inulin; O3, oolong tea with 9% inulin.

Sample	Colour Parameters			
	L *	a *	b *	ΔE
C	91.35 ^d^ ± 0.17	−1.28 ^a^ ± 0.06	10.10 ^a^ ± 0.05	-
C1	89.63 ^b^ ± 0.03	−1.18 ^a^ ± 0.05	10.23 ^a^ ± 0.10	1.73 ± 0.18
O	90.00 ^b^ ± 0.28	−0.14 ^c^ ± 0.03	12.16 ^bc^ ± 0.12	2.72 ± 0.10
O1	90.75 ^c^ ± 0.15	−0.55 ^b^ ± 0.07	12.06 ^b^ ± 0.09	0.88 ± 0.30
O2	89.07 ^a^ ± 0.26	−0.25 ^c^ ± 0.04	12.34 ^bc^ ± 0.28	0.97 ± 0.16
O3	88.62 ^a^ ± 0.11	−0.20 ^c^ ± 0.12	12.61 ^c^ ± 0.22	1.45 ± 0.39

Values are mean ± SD (*n* = 3), a, b, c, d Values followed by the same letter within a column do not differ significantly according to Tukey’s test (*p* < 0.05).

## Data Availability

Data is contained within the article and supplementary materials to the article.

## Supplementary Materials

The following are available online at https://www.mdpi.com/article/10.3390/foods10061242/s1, Table S1: Table of sensory attributes and their definitions for the profiling of yoghurts, Table S2: Results of sensory expert (*n* = 20) evaluation of the following yoghurts: C, control; C1, control with 6% inulin; O, oolong tea; O1, oolong tea with 3% inulin; O2, oolong tea with 6% inulin; O3, oolong tea with 9% inulin, Table S3: Results of the semi-consumer (*n* = 30) assessment of acceptability of and willingness to buy the following yoghurts: C, control; C1, control with 6% inulin; O, oolong tea; O1, oolong tea with 3% inulin; O2, oolong tea with 6% inulin; O3, oolong tea with 9% inulin.
