# Supplementary material for: The Sensory Quality and the Textural Properties of Functional Oolong Tea-Infused Set Type Yoghurt with Inulin
Source: Foods. 2021 May 29;10(6):1242. doi: 10.3390/foods10061242 (PMC8229014; doi:10.3390/foods10061242)
Supplement: Supplementary file 1 [file foods-10-01242-s001.zip › foods-1228695-supplementary.pdf]

foods

MDPI

Supplementary Materials

Table S1. Table of sensory attributes and their definitions for the profiling of yoghurts.

Sensory attributes	Definitions of attributes with the edge terms
Odour	
**Sweet odour**	No definition required for basic odour quality (none - very intensive)
**Sour odour**	No definition required for basic odour quality (none - very intensive)
**Yoghurt odour**	No definition required for basic odour quality (none - very intensive)
**Milky odour**	Characteristic odour of milk (none - very intensive)
**Fat odour**	Characteristic odour of fat milk (none - very intensive)
**Peach odour**	Characteristic odour of peach fruit (none - very intensive
**Nectar odour**	Characteristic odour of nectar and flowers (none - very intensive)
**Citrus odour**	Characteristic odour of citrus fruits (none - very intensive)
Appearance perceived visually	
**Colour intensity**	Visual aspect of colour intensity (light white - dark cream)
**Shine surface**	Visual presence of shine of the surface (mat – shiny)
**Whey presence**	Visual presence of whey in the product's surface and its amount (none - a lot of)
**Visual smoothness**	Visual evaluation of the degree of smoothness of the product surface (rough – smooth)
**Adhesiveness**	Visual evaluation of product density measured by the resistance of the teaspoon (thin – thick)
**Filling the teaspoon**	Visual assessment of the filling of the teaspoon by placing the product on it (flat – conical)
**Consistency uniformity**	visual assessment of the perceived consistency uniformity of the product (not uniform – uniform)
Texture/consistency perceived in the mouth	
**Smoothness in the mouth**	Mouth evaluation perception of product smoothness (rough – smooth)
**Melting**	Mouth assessment perception of product melting (much delayed – immediate)
**Creaminess**	Mouth evaluation perception of creaminess connected with melting (thin – creamy)
**Thickness**	Mouth evaluation perception of product density (thin – thick)
**Firmness**	Mouth evaluation perception of product firmness (loose – firm)
**Yield stress**	Mouth assessment perception of product viscosity (low viscosity- high viscosity)
**Fat film**	Mouth evaluation perception of fatty film (none - very intensive)
Taste/flavour	
**Sweet taste**	No definition required for basic taste quality (none - very intensive)
**Sour taste**	No definition required for basic taste quality (none - very intensive)
**Bitter taste**	No definition required for basic taste quality (none - very intensive)
**Astringent**	Astringency perception in the mouth (none - very intensive)

**Yoghurt flavour**	Characteristic flavour of yoghurt (none - very intensive)
**Milky flavour**	Characteristic flavour of milk (none - very intensive)
**Quark flavour**	Characteristic flavour of fresh cheese (none - very intensive)
**Peach flavour**	Characteristic flavour of peach fruit (none - very intensive)
**Nectar flavour**	Characteristic flavour of nectar and flowers (none - very intensive)
**Body**	Body defines harmonization of all evaluated positive attributes (incomplete – full)
**Overall quality**	Overall quality defines the overall impression of harmony of the positive attributes present, with little or no negative attributes (low–high)

Table S2. Results of sensory expert (n=20) evaluation of following yoghurts: C-control, C1-control with 6% inulin, O-oolong tea, O1-oolong tea with 3% inulin, O2-oolong tea with 6% inulin, O3-oolong tea with 9% inulin.

**Attributes**	**Yoghurt samples**					
	**C**	**C1**	**O**	**O1**	**O2**	**O3**
			**Odour**			
**Sweet odour***	1.1a	1.3a	2.5b	2.6b	3.0b	3.1b
**Sour odour***	3.1b	2.9b	1.9a	1.9a	1.5a	1.6a
**Yoghurt odour***	4.7b	4.3b	3.0a	3.0a	2.6a	2.6a
**Milky odour***	4.1b	4.2b	2.8a	2.7a	2.8a	2.5a
**Fat odour**	2.7a	3.1a	2.5a	2.3a	2.1a	1.9a
**Peach odour***	0.0a	0.0a	3.4b	3.4b	4.0b	3.9b
**Nectar odour***	0.0a	0.0a	3.7b	3.4b	4.2b	4.5b
**Citrus odour***	0.0a	0.0a	1.2bc	1.1bc	1.3c	1.5c
			**Appearance perceived visually**			
**Colour intensity***	0.9a	1.7ab	3.0c	2.5bc	1.9ab	1.8ab
**Shine surface**	7.8a	7.0a	7.8a	7.7a	7.8a	7.7a
**Whey presence***	2.2a	1.4a	3.8b	3.4b	2.3a	1.6a
**Visual smoothness**	7.6a	7.5a	7.7a	7.6a	7.8a	7.7a
**Adhesiveness**	7.3ab	7.5b	6.8a	7.2ab	6.9ab	7.5b
**Filing the teaspoon**	7.2a	7.4a	6.6a	7.1a	7.2a	7.3a
**Consistency uniformity**	7.2ab	7.2ab	7.4ab	6.8a	7.6ab	7.8b
			**Texture/consistency perceived in the mouth**			
**Smoothness in the mouth**	7.3a	7.6a	7.5a	7.2a	7.7a	7.7a
**Melting**	6.2a	6.8ab	7.1b	6.8ab	7.0ab	7.0ab
**Creaminess***	4.5a	5.8ab	5.6ab	6.6b	6.4b	6.2b
**Thickness in the mouth**	6.7a	6.9a	6.0a	6.4a	6.0a	6.8a
**Firmness***	6.1a	7.0b	5.9a	6.6ab	5.7a	6.1a
**Yield stress**	2.5ab	2.9ab	2.1a	2.9ab	3.0b	2.4ab

**Fat film**	2.0a	2.9b	2.5ab	2.9b	2.5ab	3.0b
			**Taste/flavour**			
**Sweet taste***	0.9a	3.9de	1.6ab	2.5bc	3.0cd	4.2e
**Sour taste***	4.9c	2.7a	4.0bc	3.7ab	3.9bc	3.0ab
**Bitter taste***	0.2a	0.1a	1.2b	0.9ab	1.3b	0.8ab
**Astringent taste**	0.9ab	0.5a	1.2ab	1.3b	1.1ab	0.9ab
**Yoghurt flavour***	5.4c	4.9bc	3.6a	3.9ab	4.1ab	3.7a
**Milky flavour***	4.6b	4.2b	2.7a	3.2a	2.7a	2.4a
**Quark flavour**	2.7a	2.9a	1.8a	2.4a	2.4a	2.0a
**Peach flavour***	0.1a	0.2a	2.8b	2.7b	3.8b	3.7b
**Nectar flavour***	0.1a	0.2a	2.9b	2.7b	3.5b	3.9b
**Body**	5.5ab	5.8ab	4.7a	5.4ab	6.5b	6.0b
**Overall quality**	5.6a	6.5a	5.4a	5.5a	6.3a	6.2a

*Mean values differ significantly (p ≤0.05).

Table S3. Results of the semi-consumer (n=30) assessment of acceptability of the following yoghurts: C-control, C1-control with 6% inulin, O-oolong tea, O1-oolong tea with 3% inulin, O2-oolong tea with 6% inulin, O3-oolong tea with 9% inulin, and willingness to buy them.

	**C**	**C1**	**O**	**O1**	**O2**	**O3**
**Appearance**	7.2a	7.4a	6.8a	6.7a	7.5a	7.4a
**Odour**	6.5a	7.0a	6.8a	6.6a	6.8a	6.8a
**Consistency**	6.2a	6.9a	6.4a	6.0a	6.9a	6.8a
**Flavour**	5.4a	6.1a	6.0a	5.7a	6.2a	6.4a
**Overall acceptability**	5.8a	6.2a	5.7a	5.9a	6.5a	6.6a
**Willingness to buy**	5.9a	6.0a	5.8a	5.7a	6.4a	6.6a

*Mean values differ significantly (p ≤0.05).
